# Towards Polypharmacokinetics: Pharmacokinetics of Multicomponent Drugs and Herbal Medicines Using a Metabolomics Approach

**DOI:** 10.1155/2013/819147

**Published:** 2013-03-14

**Authors:** Ke Lan, Guoxiang Xie, Wei Jia

**Affiliations:** ^1^Key laboratory of Drug Targeting and Drug Delivery System of the Education Ministry, West China School of Pharmacy, Sichuan University, Chengdu 610041, China; ^2^Center for Translational Biomedical Research, University of North Carolina at Greensboro, North Carolina Research Campus, Kannapolis, NC 28081, USA

## Abstract

Determination of pharmacokinetics (PKs) of multicomponent pharmaceuticals and/or nutraceuticals (polypharmacokinetics, poly-PKs) is difficult due to the vast number of compounds present in natural products, their various concentrations across a wide range, complexity of their interactions, as well as their complex degradation dynamics *in vivo*. Metabolomics coupled with multivariate statistical tools that focus on the comprehensive analysis of small molecules in biofluids is a viable approach to address the challenges of poly-PK. This paper discusses recent advances in the characterization of poly-PK and the metabolism of multicomponent xenobiotic agents, such as compound drugs, dietary supplements, and herbal medicines, using metabolomics strategy. We propose a research framework that integrates the dynamic concentration profile of bioavailable xenobiotic molecules that result from *in vivo* absorption and hepatic and gut bacterial metabolism, as well as the human metabolic response profile. This framework will address the bottleneck problem in the pharmacological evaluation of multicomponent pharmaceuticals and nutraceuticals, leading to the direct elucidation of the pharmacological and molecular mechanisms of these compounds.

## 1. Introduction

Multicomponent herbal medicines have gained popularity as complementary interventions against a number of conditions, including metabolic diseases and cancer [1, 2]. Distinct from the pharmacology of chemical drugs (single compounds), the pharmacology of multi-component agents, including botanical-based nutraceuticals, entails a “network” approach, in which multiple compounds interact *in vivo *with multiple targets with interdependent activities to achieve an optimal effect [3, 4]. The vast number of metabolites present in natural products and their wide dynamic range are inextricable obstacles for pharmacological evaluation and nutraceutical/drug development. Furthermore, the co-existence of multiple compounds may lead to metabolic and pharmacokinetic interactions. Due to the complexity of both botanicals and biological samples (e.g., blood, urine, tissues), the analytical approaches to quantitatively measure the time-dependent concentration profiles of bioavailable plant molecules (i.e., poly-PK) are beyond the scope of traditional research. In fact, herbal medicine PK that simultaneously monitors multiple metabolites (i.e., poly-PK) has only been reported in a handful of studies [5, 6]. As a result, poly-PK has been a long-standing bottleneck in botanical-based medical and nutritional research. 

The traditional approach to understand the pharmacology of a multi-component agent is to study the effects of single components on single biological reactions, enzymes, genes, and so forth, and gradually assemble the findings into a whole picture. However, assembling the results obtained from such a reductionist approach to achieve a system understanding of a concerted pharmacological intervention has proven impractical [7]. In fact, the results of such attempts failed to accurately capture the complex pharmacokinetic behavior of herbal medicines. Additionally, the PK of a given compound in a multi-component assay may be significantly different from that in a single-compound assay due to drug-drug interactions. With such a complex network involving a large number of variables, it is technically challenging to identify each metabolite that changes significantly in the global metabolite pool, and to assess the human (endogenous) biochemical responses to exposure to these exogenous (xenobiotic) compounds. 

The complicated metabolic fate of chemicals in the human body is primarily determined by the chemical's structure and greatly varies according to dose, routes of exposure, inter- and intraindividual genetic differences, gut microbiota, diet, life style, and environment, as well as other xenobiotics intentionally or unintentionally present. In order to simplify these intricate factors, a knowledge-based engine that sequentially manipulates each factor *in silico* for prediction, coupled with *in vitro* screening and identification, and *in vivo *validation is deployed for xenobiotic metabolism studies [8]. However, because cross-platform extrapolations are not always the reliable or robust [9], the *in vivo* xenobiotic metabolite studies require special attention; these compounds are often intermixed against the background of endogenous metabolites in biological samples. Recent advances in liquid, and gas chromatography (LC and GC) and capillary electrophoresis (CE) coupled with mass spectrometry (LC-MS, GC-MS, and CE-MS) and nuclear magnetic resonance (NMR) have made it possible to simultaneously detect various small-molecule metabolites [10]. 

Metabolomics is the study of the metabolome, the complete set of endogenous metabolites in a biological sample. In context of systems biology [11, 12], metabolomics is favored not only for decoding how a biological system responds to a stimulus by identifying the most significantly affected endogenous metabolites and their metabolic pathways, but also resolving the relationships between endogenous and xenobiotic metabolic processes [13]. Metabolomics techniques have offered xenobiotic studies a novel way to differentiate the exogenous from the endogenous by untangling the interactive metabolic pathways for both. Capable of simultaneously analyzing hundreds and thousands of variables, metabolomics is uniquely suited to develop a new generation of PK platforms that can measure multiple nutraceutical components *in vivo*, as well as identify characteristic metabolic alterations in humans exposed to nutraceuticals [14, 15]. Along these lines, utilizing metabolomics to study herbal medicine efficacy and toxicity has been a key focus of recent herbal and pharmaceutical research [16–20]. Recently, we proposed an integrated metabolomic profiling strategy for PK and PD studies of multi-component drugs using tandem mass spectrometry (MS/MS) [21] and conducted a proof-of-concept poly-PK study of Pu-erh tea intervention in humans based on metabolomics [6]. 

Metabolomics might assume the new mission to model the variations of both exogenous and endogenous metabolites through a given biological system, which has vital implications in both systems biology and multi-component herbal medicine. The aim of this review is to discuss recent progress in the characterization of poly-PK and metabolism of multi-component xenobiotic agents such as compound drugs, dietary supplements, and herbal medicines using metabolomics. We also propose a research framework that integrates the dynamic concentration profile of bioavailable xenobiotic molecules that result from *in vivo* absorption, and hepatic and gut bacterial metabolism, as well as the human metabolic response profile. 

## 2. Metabolomics in Xenobiotic Intervention Study

**Figure 1:**
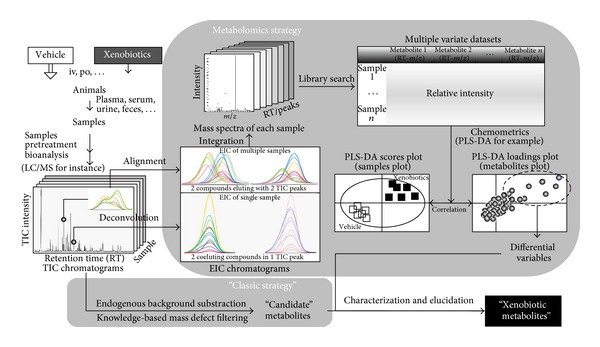
LC-MS protocol for xenobiotic intervention studies with and without the incorporation of metabolomics. Iv: intravenous; po: oral; EIC: extracted ion chromatogram; PLS-DA: partial least squares-discriminant analysis; TIC: total ion chromatogram.

Metabolomics enables the profiling of a vast number of small-molecule metabolites in an organism or a biological system as a whole, and helps understand how these metabolites respond to a stimulus, highlighting the most relevant potential biomarkers. When the stimulus is a xenobiotic intervention, metabolomics techniques are employed to profile the metabolite pool with and without the xenobiotic and highlight the differential variables to identify the xenobiotic metabolites. A comparison of a typical protocol for xenobiotic intervention studies using LC-MS-based metabolomics and the classical LC-MS-based workflow is illustrated in Figure 1. The major difference between the two methods is the data processing procedure to differentiate exogenous metabolites against the endogenous metabolite background. The metabolomics strategy employs multivariate data pretreatment methods [22] (e.g., deconvolution, alignment, integration) coupled with multivariate analyses [15, 23] (e.g., principle component analysis (PCA), partial least squares discriminant analysis (PLS-DA)). In contrast, the classical strategy utilizes arbitrary endogenous background subtraction and/or knowledge-based mass defect filtering, which takes advantage of known mass changes between the parent ion and metabolite ions [24].

Such a metabolomics-based protocol was first proposed in 2003 by Plumb and colleagues in an attempt to find the *in vivo* metabolites of GSK-X, the structure of which was unknown to the investigators [29]. Shortly after, another metabolomics-based protocol identified several new metabolites of citalopram [30]. Consequently, Dr. Gonzalez from National Cancer Institute at the National Institute of Health expanded LC-MS-based metabolomics for xenobiotic metabolism studies by developing various strategies, including (1) identification of metabolites by metabolomic analysis with or without xenobiotic intervention (Figure 1, and [31]), (2) validation of metabolites through metabolomic comparison between xenobiotic treatments with or without stable isotope label in order to highlight the xenobiotic metabolites in contrast to the endogenous ones altered by xenobiotic intervention, (3) identification of metabolic pathways through metabolomic comparison of wild-type and genetically-modified mice, and (4) identification of polymorphisms of genes encoding human xenobiotic metabolism enzymes (XMEs) that may be responsible for adverse drug reactions [31]. Two recent reviews address the technical issues and progress in this field [32, 33].

Compared to the classical methods in xenobiotic intervention studies, metabolomics strategies have clear advantages, firstly, in the capacity of handling a great number of variables—which allows a shift in purpose from targeting xenobiotic metabolites to profiling the complete set of metabolites in biological samples—and secondly, in the unbiased selection of variables that are significantly altered—which facilitates the discrimination of xenobiotic metabolites from endogenous ones. Due to these advantages of metabolomic strategies, a novel, evidence-based methodology that starts with mapping *in vivo* metabolites and leads to further *in vitro* characterization has been established, which is in stark contrast to the classic, knowledge-based methodology that starts with *in vitro* identification to *in vivo* characterization, and is frequently muddled by inconsistent results. Such a methodological shift obviates potential false-negative results that usually result from traditional empirical strategies, and significantly deepens our insight into the *in vivo* metabolic map of xenobiotics in at least two ways. First, metabolomics captures *in vivo* xenobiotic metabolites that are otherwise easily overwhelmed by the endogenous background. Examples of such metabolites are those with unexpected skeleton biotransformation or those at extremely trace levels with potential clinical significance and wide usage in the general population. Second, the consideration of genetic and environmental factors affecting the metabolism of xenobiotics in metabolomics studies facilitates the *in vivo* identification of metabolic pathways and factors affecting them.

## 3. Current Status of Single Xenobiotic Intervention Studies with Metabolomics Strategy

To date, metabolomics techniques have been employed in metabolism studies of dozens of single xenobiotics with diverse chemical structures (Table 1), including GSK-X [29], citalopram [30], aminoflavone [34], arecoline/arecaidine [35], PhIP [36], arecoline 1-oxide [37], ferulic and sinapic acids [38], acetaminophen [39–42], dextromethorphan [43], melatonin [44, 45], vitamin E [46], fenofibrate [47, 48], tolcapone [49], cyclophosphamide/ifosfamide [50], tipranavir [51], nefazodone [52], valproic acid [53], ritonavir [54], pulegone/clozapine [42], thioTEPA [55], isoliquiritigenin [56], ethanol [57], and procainamide [58]. Remarkably, majority of these studies were carried out *in vivo*. It has been proposed that such metabolomics strategies can be used to study any organic molecule in any animal model, provided that the protocol is carefully designed, controlled, and executed [32]. In our opinion, a metabolomics approach is especially useful for studying xenobiotics with known toxicities and multiple metabolite endpoints predicted by their chemical structures. As a result, the insights from metabolomics extend our knowledge of how a xenobiotic affects human body from a list of its major metabolites to the complete *in vivo* metabolite map. The detailed information about multiple bioactive metabolites may significantly advance our understanding of various and rare adverse drug reactions in the general population.

## 4. Progress and Challenges of Herbal Exposure Studies

Multicomponent drugs and herbal medicines have an extremely complicated and highly variable chemical composition and introduce multiple xenobiotics into the human body. The metabolism of these xenobiotics may provide insights as to why and how they work by revealing changes* in vivo* due to herbal exposure. However, the unclear metabolic fate of herbal medicines causes significant limitations in understanding the efficacy and toxicity of these substances. Compared to single xenobiotics, of which there are significant advances in terms of metabolic fate, multi-component herbal medicines present a challenge due to their complex nature. 

**Figure 2:**
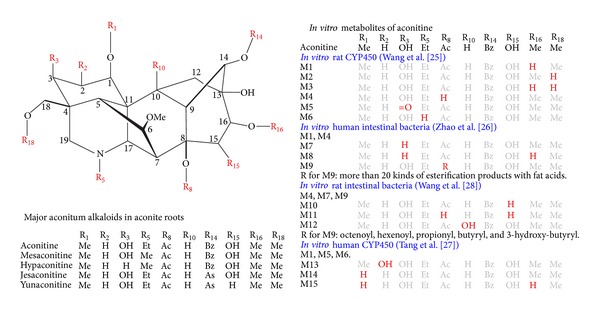
Major aconitum alkaloids in aconite roots and *in vitro* metabolites of aconitine [25–28]. Me: methyl group; Et: ethyl group; Ac: acetyl group; Bz: benzoyl group; As: anisoyl group.

For the development of *in vivo* xenobiotic metabolism protocols for multi-component drugs and herbal medicines, there are several major obstacles: (1) the difficulty to determine and standardize the chemical composition of multi-component drugs and herbal medicines, (2) the overlap between the chemical composition of herbal medicines with that of daily diets, (3) the extensive microbial-mammalian co-metabolism in the gut of herbal components [67] that vary by species (e.g., see the *in vitro* metabolites of aconitine, illustrated in Figure 2 [25–28]), (4) the difficulty of differentiating exogenous metabolites from the endogenous background 1–3, and (5) the difficulty of resolving the intercrossed metabolic pathways of different herbal components that share similar chemical skeletons (for an example, see aconitum alkaloids in aconite roots, illustrated in Figure 2). Therefore, current research to elucidate the xenobiotic metabolism of herbal exposures is still in its infancy, exploring and developing solutions to these challenges.

### 4.1. Classic Strategy

The strategies and techniques for *in vitro* and *in vivo* herbal exposure studies following classic, knowledge-based methodologies have recently been discussed [68, 69]. With the advances in bioanalytical methods, many metabolites of herbal interventions were successfully captured. For example, a recent study of the herbal supplement Danggui Buxue Tang detected and identified 142 metabolites from bile and plasma samples [70]. However, from the efforts to map the metabolites of single xenobiotics, it has become clear that the predictive and provisional approaches used in classic strategies can be inaccurate and lack reproducibility.

Nevertheless, two strategies have accomplished noteworthy advances in metabolite mapping of xenobiotics. One of these strategies was developed from the identification of individual metabolites of licorice [71]. In fact, more than 60 metabolites were identified and PK profiles of 55 were obtained [72]. This strategy was robust and managed to reveal intraherb metabolic interactions; however, it requires prior knowledge of herbal components and is time consuming. The other strategy was based on matching the “chemicalome” (referring to LC-MS data of the tested herbal medicine) to the “metabolome” (referring to LC-MS data including tentative herbal metabolites acquired by endogenous background subtraction) upon the injection of the herbal medicine Mailuoning [73]. Matching was performed by mass defect filtering, which incorporated accurate mass changes of 62 types of metabolic reactions. Using this approach, 143 metabolites were identified in urine. This strategy exhibited clear superiority in labor-saving predictions; however, the endogenous background subtraction was arbitrary, introducing false positives and excluding false negative results.

### 4.2. Metabolomics-Based Strategy

The Recent advances in metabolomics encouraged researchers to start exploring the effects of multi-component drugs/herbal medicines using metabolomics strategies. To the best of our knowledge, the first attempt of this kind was the NMR-based metabolomics study on human nutritional intervention of grape/wine extract reported by van Velzen and colleagues in 2008 [63]. This study aimed to distinguish between-subject and within-subject variations in metabolomic data matrices using multivariate paired data analyses corresponding to a crossover design. Since its major results concerned metabolites of polyphenols, the integration of PK modeling into metabolomics was developed in a later study by the same group [64]. The proposed multivariate paired data analysis [74] and dynamic metabolomic data analysis [75], in combination with study designs that tackle the challenges arising from dietary metabolite background and microbial-human cometabolism in the gut [76], have collectively coined the term “nutrikinetics,” a new discipline that combines cutting-edge technologies and new methodologies to get a complete picture of what happens to the consumed metabolites and supplements in the human body [77]. Nutritkinetics relies heavily on metabolomics biostatistical analyses. To date, there have been a number of studies on dietary exposure incorporating metabolomics, which were summarized in a recent review on nutrimetabolomic strategies [78]. 

The metabolomics-based strategies using LC-MS have clear advantages, as they can handle a great number of variables and large datasets, enable graphic representation of metabolism-related sample classification, and identify drug compounds and significant drug metabolites. In fact, several publications reported the use of metabolomics to capture both drug-derived and drug-induced metabolites [66] and the alterations of metabolites in urine after cocoa powder consumption [60] (Table 1). Despite the fact that metabolomics can be employed in PK studies of herbal medicines, it is almost impossible to detect the complete pool of metabolites in a biological sample the existing analytical platforms [79]. Indeed, categorizing the origins of altered metabolites as a result of herbal intervention still present with significant challenges [21], including the categorization of: (1) intact herbal components absorbed into circulation, (2) xenobiotic metabolites processed by hepatic enzymes and gut microbes, and (3) endogenous metabolites that are significantly altered in response to the intake of plant-derived compounds. 

**Figure 3:**
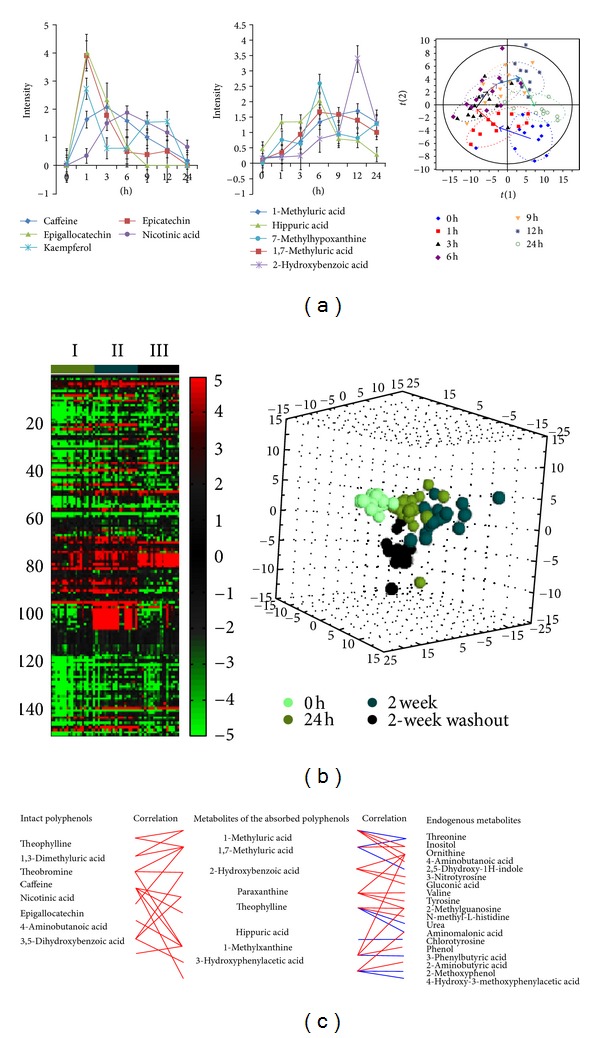
Dynamic concentration profile of bioavailable Pu-erh tea polyphenols due to *in vivo* absorption, and hepatic and gut bacterial metabolism, as well as the human metabolic response profile. (a) Urine concentration-time course of some representative substances, intact polyphenols, metabolites of the absorbed polyphenols, and altered endogenous metabolites, after Pu-erh tea intake; (b) effect of Pu-erh tea intake on human urine metabolite endpoints. (left panel) Heatmap showing differences in altered endogenous metabolites detected from the metabolome after Pu-erh tea intake (postdose) as compared to predose metabolome. (I) metabolomic changes at 24 h postdose relative to predose; (II) 2-week postdose versus predose; (III) 2-week washout versus predose. Each cell in the heat map represents the fold change between the two time points (e.g., postdose versus the predose) for a particular metabolite. (right panel) 3D PCA scores plot of urinary metabolic profiles at predose, 24 h postdose, 2 week postdose, and 2 week washout postdose; (c) correlation of intact polyphenols, metabolites of the absorbed polyphenols, and altered endogenous metabolites in response to Pu-erh tea exposure. The relationships among the three groups of compounds were visualized in the form of correlation maps, which are displayed by red (positive) or blue (negative) lines.

We believe that categorization of these metabolites in herbal intervention studies will benefit more from developments in multivariate analyses than in advances in analytical techniques. To this end, we have proposed a multivariate similarity analysis to categorize the altered metabolites as a result of herbal intervention [21]. Recently, we used this strategy to analyze the metabolic fates of Pu-erh tea polyphenols in humans [6]. Urine samples were collected at baseline and at different time periods for 6 weeks. Volunteers ingested Pu-erh tea daily, followed by a wash-out phase during these 6 weeks. The urine samples were analyzed using ultraperformance liquid chromatography-quadrupole time of flight mass spectrometry (UPLC-QTOFMS) and gas chromatography-time of flight mass spectrometry (GC-TOFMS). The resulting dataset composed of 6,028 detected features was subjected to univariate statistical analysis, yielding 2,476 and 176 altered variables from UPLC-QTOFMS and GC-TOFMS were highlighted, respectively (*P* < 0.05). Using multivariate similarity analysis to compare the altered variables to the plant metabolome or the predose human metabonome, 19, 26, and 118 metabolites were categorized as intact tea polyphenols, metabolites of the absorbed polyphenols, and endogenous metabolites altered due to tea intake, respectively. The subsequent dynamic correlation analysis produced, for the first time, a correlation between the herbal metabolic network and endogenous metabolism (Figure 3).

As discussed above, despite great challenges, a strategy based on metabolomics has great potential capabilities to discover not only the components in herbal exposure and their *in vivo *metabolites, but also the endogenous metabolites altered by herbal interventions. In this regard, the metabolic shells covering the interactions between multiple xenobiotics and human biological system will be shucked in future metabolomics incorporating xenobiotic metabolism.

## 5. Proposed Research Framework

The essence of balances in traditional Chinese medicine and herbal medicines align very closely with the core concepts of systems biology [80], which aims to theoretically and experimentally describe homeostasis of a biological system and its allostasis with an “-omics” approach [81, 82]. Metabolomics, as a “top-down” -omics technology, evaluating small-molecule metabolites as the ultimate downstream products of genomic, transcriptomic, and/or proteomic perturbations, is encouraging herbal medicine researchers to tackle existing issues in the field from chemistry to biology. However, besides its phytochemical applications, which focus on the quality assessments of botanic products [83–85], metabolomics in herbal medicine mostly addresses biomedical implications for the endogenous metabolism [86–90], while paying little attention to the metabolism of the xenobiotics, which is tightly linked to and has important implications for human metabolic networks.

**Figure 4:**
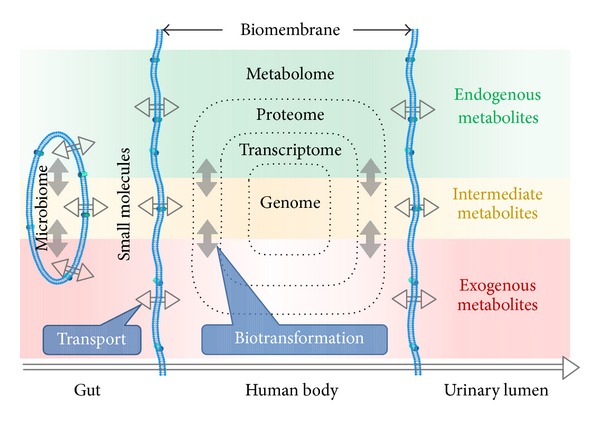
Research framework to integrate the dynamic concentration profile of bioavailable xenobiotic molecules due to *in vivo* absorption and the hepatic and gut bacterial metabolism, as well as the human metabolic response profile.

Based on the above and guidance from related studies, we propose a research framework to integrate the dynamic concentration profile of bioavailable xenobiotic molecules due to *in vivo* absorption and the hepatic and gut bacterial metabolism,as well as the human metabolic response profile in Figure 4. Our framework highlights the flux of small-molecule metabolites through the human body with gut as the major entrance and urinary lumen as the major exit points. Recent multicompartmental (plasma, urine, and caecal contents) metabolomic work depicted this framework in a smaller scale [91]. In our proposed model, the human body has evolved as a superorganism encompassing the genome, transcriptome, proteome, metabolome, and symbiotic gut microbiome, that latter coexisting with the host depending on genetic and environmental factors [92–96]. It is of special importance for the gut microbiome and the host to maintain ceaseless substance exchange, in which the small-molecule metabolites can permeate the biomembrane barrier. These metabolites can be divided into endogenous and exogenous metabolites according to their predicted sources; however, the distinction between endogenous and exogenous is not so clear due to the gut microbiome. As a result, metabolites with unclear origins can be classified in an intermediate group. For example, endogenous ethanol is not a metabolite of human metabolism, but a byproduct of gut microbes [97, 98], which is associated with nonalcoholic fatty liver disease [99]. In all, biotransformation (metabolism) and transportation are essential to the metabolic network of host and microbial interactions to dispose of endogenous and exogenous metabolites.

The host-gut microbial metabolic networks for endogenous and exogenous metabolites are highly interactive but with subtle differences in metabolic fates. It is beyond doubt that the superorganism's metabolism has to strike a balance between the uptake of essential nutrients and the elimination of superfluous metabolites to maintain homeostasis, yielding a relatively stable internal environment of endogenous metabolites. The involvement of gut microbiota in endogenous metabolic pathways [100–104], as a matter of fact, has given rise to interactive host-microbiota metabolic, signaling, and immuneinflammatory axes that physiologically connect the gut, liver, muscle, and brain [105]. On the other hand, the superorganism cannot be too lenient with regard to exogenous metabolites, maintaining allostasis to achieve a stable internal environment by eliminating exogenous metabolites. Gut microbiota supplements the human metabolic system with xenobiotics [106] by directly taking part in their metabolism, especially in the metabolism of natural products such as flavonoids, saponins [67], polyphenols [76], and alkaloids [26, 28], as well as the metabolism of exogenous metabolites, such as the competitive sulfation of acetaminophen and p-cresol [107], and the regulation of XMEs [108, 109]. Based on the distinction between the metabolic fates of exogenous and endogenous metabolites, trend analysis was proposed as an alternative to metabolite discovery [110].

Therefore, the superorganism has evolved a highly delicate and interactive host-gut microbiota metabolic network to keep its homeostasis and trigger allostasis. Following our framework, in combination with advances in genomics, transcriptomics, proteomics, and metagenomics, future metabolomics strategies will contribute to our understanding of how the balance emphasized in comprehensive herbal medicine is correlated with the homeostasis and allostasis addressed in systems biology.

## 6. Conclusion

In conclusion, recent studies suggest that metabolomics coupled with multivariate statistical tools can offer an alternative to address the challenges of the determination of PK of multi-component pharmaceuticals and/or nutraceuticals. We believe that the use of a metabolomics strategy in pharmacological studies has important advantages over conventional approaches for multi-component therapeutics. The integration of metabolomics to study the metabolism of xenobiotics will unravel the complicated variations in multiple metabolites of endogenous and exogenous origin within the host-gut microbial symbiotic network, and tease out the underlying mechanisms of homeostasis and allostasis in terms of systems biology. Progress in this field will definitely help the poly-PK studies of multi-component herbal medicines to assess their efficacy. Acquisition of a complete and dynamic panel of pharmacokinetic parameters for multi-component dosage regimens to achieve desired therapeutic efficacies is essential to minimize toxicity, reduce overdosing and drug complications, keep healthcare costs at a minimum, and ultimately, increase patient compliance and quality of life. 

## Figures and Tables

**Table 1:** Summary of PK studies of single chemicals and multicomponent drugs or herbal medicines using metabolomics.

Substance	Type of biological sample	Bioanalysis technique	Multivariate analysis method	Reference
GSK-X	Rat urine	LC-TOFMS	PCA, Ward's HCA	[29]
Citalopram	Rat urine	LC-MS	PCA, PLS, PARAFAC, N-PLS	[30]
Ferulic acid and sinapic acid	Rat urine	HPLC-QTOFMS	PLS-DA	[38]
Fenofibrate	Rat urine	UPLC-QTOFMS	PLS-DA	[47]
Isoliquiritigenin	Rat urine	UPLC-QTOFMS	PCA, PLS-DA	[56]
Tolcapone	Rat urine	UPLC-QTOFMS	PCA	[49]
Acetaminophen	Rat urine	UPLC/MS, NMR	PCA, PLS-DA	[41]
Valproic acid	Mouse urine	LC-MS	PCA	[53]
ThioTEPA	Mouse urine	UPLC-QTOFMS	OPLS	[55]
NSC686288 (aminoflavone)	Mouse urine	UPLC-QTOFMS	PCA	[31, 34]
Ethanol	Mouse urine	UPLC-QTOFMS	PLS-DA, OPLS	[57]
Arecoline and arecaidine	Mouse urine	UPLC-QTOFMS	PCA, PLS-DA	[35]
(±)-Arecoline 1-oxide	Mouse urine	UPLC-QTOFMS	PCA	[37]
PhIP*	Mouse urine	UPLC-QTOFMS	PCA	[36]
Acetaminophen	Mouse urine	UPLC-QTOFMS	PCA	[39]
Cyclophosphamide/ifosfamide	Mouse urine	UPLC-QTOFMS	OPLS	[50]
Vitamin E	Mouse urine	UPLC-TOFMS	PCA, PLS-DA	[46]
Melatonin	Mouse urine	IPLC-TOFMS, LC-MS/MS	PCA, OPLS	[44]
Tipranavir	Mouse urine, feces, tissue	UPLC-TOFMS	PCA, OPLS-DA	[51]
Ritonavir	Mouse urine and feces	UPLC-TOFMS	OPLS-DA	[54]
Procainamide	Mouse and human urine	UPLC-QTOFMS	PCA, PLS-DA	[58]
Fenofibrate	Monkey urine	UPLC-QTOFMS	PLS-DA	[48]
Pulegone/clozapine	Mouse liver	UPLC-TOFMS	PCA, OPLS-DA	[42]
Dextromethorphan	Human urine	LC-MS/MS	PCA, OPLS-DA	[43]
3,4-Dehydro-debrisoquine	Human urine	UPLC-QTOFMS	OPLS	[31, 59]
Cocoa powder	Human urine	HPLC-QTOFMS	PLS-DA, two-way HCA	[60]
Almond skin extract	Human urine	LC-QTOFMS	PCA, OPLS-DA	[61]
Pu-erh tea	Human urine	UPLC-QTOFMS, GC-TOFMS	PCA, OPLS-DA	[6, 62]
A mix of wine extract and grape juice extract	Human urine	^ 1^H NMR	ANOVA-PCA/PLS	[63]
Dried black tea extract and red grape extract	Human urine	^ 1^H NMR	ANOVA-PCA/PLS	[64]
Chamomile tea	Human urine	^ 1^H NMR	PCA, PLS, OSC	[14]
Dark chocolate	Human urine, plasma	^ 1^H NMR, LC-MS, LC-MS/MS	PCA, OPLS-DA	[65]
Nefazodone	NADPH-supplemented human liver microsomal incubation samples	LC-MS	PCA	[52]
Rifampicin, phenobarbital, and CITCO**	Human hepatocytes	UPLC-TOFMS	PCA, OPLS, SUS plot	[66]

*2-Amino-1-methyl-6-phenylimidazo[4,5-b]pyridine.

**6-(4-Chlorophenyl)imidazo[2,1-b][1,3]thiazole-5-carbaldehydeO-3,4-dichlorobenzyl) oxime.

LC-MS: liquid chromatography-mass spectrometry; UPLC-QTOFMS: ultraperformance liquid chromatography-quadrupole time of flight mass spectrometry; NMR: nuclear magnetic resonance; GC-TOFMS: gas chromatography-time of flight mass spectrometry; PCA: principle component analysis; PLS: partial least squares; OPLS-DA: orthogonal partial least squares-discriminant analysis; DAPARAFAC: parallel factor analysis; N-PLS: multilinear partial least squares; OSC: orthogonal signal correction.
